# Correction: Intragastric exposure to titanium dioxide nanoparticles induced nephrotoxicity in mice, assessed by physiological and gene expression modifications

**DOI:** 10.1186/1743-8977-10-51

**Published:** 2013-10-09

**Authors:** Suxin Gui, Xuezi Sang, Lei Zheng, Yuguan Ze, Xiaoyang Zhao, Lei Sheng, Qingqing Sun, Zhe Cheng, Jie Cheng, Renping Hu, Ling Wang, Fashui Hong, Meng Tang

**Affiliations:** 1 Medical College of Soochow University, Suzhou 215123, China; 2 Key Laboratory of Environmental Medicine and Engineering, Ministry of Education, School of Public Health, Southeast University, Nanjing 210009, China; 3 Jiangsu key Laboratory for Biomaterials and Devices, Southeast University, Nanjing 210009, China

## Correction

After publication of this article [1], the authors became aware of the fact that the original version of this article missed equal contributor and corresponding author information.
